# Editorial: Proteomic Approaches to Unravel Mechanisms of Resistance and Immune Evasion of Bacterial Pathogens

**DOI:** 10.3389/fmed.2022.945264

**Published:** 2022-07-07

**Authors:** Eva Torres-Sangiao, Nelson C. Soares, Xiaoyun Liu

**Affiliations:** ^1^Clinical Microbiology Lab, University Hospital Marqués de Valdecilla, Santander, Spain; ^2^Instituto de Investigación Sanitaria Marqués de Valdecilla (IDIVAL), Santander, Spain; ^3^Sharjah Institute of Medical Research, University of Sharjah, Sharjah, United Arab Emirates; ^4^Department of Medicinal Chemistry, University of Sarjah, Sharjah, United Arab Emirates; ^5^Department of Microbiology, School of Basic Medical Sciences, Peking University Health Science Center, Beijing, China

**Keywords:** quantitative mass spectrometry, antibiotic resistance, proteomics, immunoproteomics, biomarkers, protein-protein interactions, bacterial virulence, antimicrobials

Our knowledge about the mechanisms involved in antimicrobial resistance (AMR) and immune evasion has evolved greatly over the past decade, since we are now better able to understand them, especially AMR, thanks to next-generation sequencing. In addition, during the pandemic we were able to learn about immune evasion and microbial evolution *in situ*. Most pathogens are able to modulate and manipulate the host immune system to avoid the immune response. However, the lack of new and efficient treatments will lead to untreatable infections that become more widespread, with increased morbidity and mortality. Therefore, immunomodulation approaches, such as adjuvants, biological therapies, and vaccines, appear to be the most promising methods to enhance an immune response. Fortunately, emerging technologies under the “*omic*” approach are helping us to understand the molecular mechanisms that interplay within the host pathogens, including AMR. The study of the host-pathogen protein interactions, their evolution over time and under different therapeutic strategies, as well as predictive models, are key to understanding complex systems and to unlocking new strategies. Here, Frontiers in Medicine presents a Research Topic on this cutting-edge theme. In this Research Topic, we collected one review, one perspective, and five original research articles, which together give an overview of the status of this field.

In the review, Torres-Sangiao et al., the authors describe different mass spectrometry strategies to investigate the interaction networks established during the infection. The authors described in-depth how protein-protein interactions reveal the mechanism involved in the molecular processes of infectious diseases through an overview of high-throughput mass spectrometry applications e.g., SWATH/DIA MS, that enable the identification of thousands of proteins in a matter of minutes. This type of in-depth analysis in a short period of time, combined with other supportive high-tech applications such as data processing and artificial intelligence, represents a unique opportunity to translate knowledge-based findings into measurable findings or facts, such as new antimicrobial biomarkers and drug targets. Certainly, the molecular mechanism of infectious diseases are not completely known and fully understood, so for it, will require the collaboration of different disciplines ranging from proteomics to molecular dynamics simulations, together with big data and deep learning.

In the context of host-pathogen interactions, virulence factors or effectors from Gram-negative bacteria may be transferred from bacterial pathogens to eukaryotic host cells by e.g., multicomponent Type III secretion systems (T3SSs). De Meyeret al. demonstrated the utility of the recently described Virotrap interactomics approach to catalog effector-host protein/protein interactions of Gram-negative *Salmonella* Typhimurium. Through this novel mass spectrometry based on Virotrap analysis, the authors showed that the arginine glycosyltransferase SseK1 was implicated in death-receptor signaling and the actin-binding cluster was significantly enriched among hits for SspH2. The authors showed that Virotrap supplements the current state-of-the-art interactomics toolkit, demonstrating a valuable means for screening host effector targets in a high-throughput way, thereby bridging the knowledge gap between effector-host interplay and pathogenesis.

Another well-known pathogen is *Escherichia coli*, that together with *Proteus* and *Enterococcus* spp. (Gram-positive bacteria) are pathogen species well-adapted to the human urogenital tract. By adhesion and multi-layered growth, these uropathogens trigger host immune responses provoking a bacterial starved off nutrients, and consequently their bacteria spreading. In addition, the biofilms formed are usually composed of multiple microorganisms that colonize the surfaces of indwelling urethral catheters, which are frequently used by neurogenic bladder patients, and cause chronic infections. However, the molecular adaptations of bacteria in catheter biofilms are not well-understood yet, promising new insights into clinical specimens. For up to 6 months, Yu et al. examined catheters from nine neurogenic bladder patients, observing that the i.v (intravenous) antibiotic treatment of these nine patients resulted in either transient or lasting microbial community perturbations. Finally, they discovered that the deactivation of the Nqr respiratory system can compromise *P. mirabilis* growth in a basic pH environment. However, to validate it as a new drug target, more studies in animal models will be necessary to gather molecular-level insights into polymicrobial biofilm metabolism and interactions.

Another matter of concern is cystic fibrosis (CF), a rare disease caused by a mutation of the *CF transmembrane conductance regulator*, a gene encoding a channel protein of the apical membrane of epithelial cells. The alteration of Na^+^ and K^+^ transport induces the accumulation of dense and sticky mucus in the patient's airways, which promotes recurrent infections. *Pseudomonas aeruginosa* (PA) is the most frequently detected bacterium involved in chronic colonization, which requires stringent antibiotic therapy that unfortunatly leads to multi-drug resistance. PA uses an adaptive mechanism to modulate and adapt itself to the new scenario through surface molecules such as efflux pumps, flagellum, pili, and other virulence factors. In the study of Montemari et al., the authors compared surface protein expression of PA multi- and pan-drug resistant strains to wild-type antibiotic-sensitive strains, isolated from the airways of CF patients with chronic colonization and recent infection, respectively. Applying a novel shaving proteomic approach, they described the adaptation processes of a large collection of PA clinical strains isolated from CF patients in early and chronic infection phases.

AMR is a well-recognized and widespread area of increasing concern, as we mentioned previously. The proteomic approach can help to generate more knowledge at the phenotypic and metabolic level. Deatherage Kaiser et al. demonstrated that proteins associated with paired AMR are significantly impacted compared to antimicrobial susceptible (AMS) strains of *Yersinia pestis* (diseases plague) and *Francisella tularensis* (etiological agent of tularemia). They hypothesized that those proteins involved in specific metabolic pathways and biological functions show altered abundance independently of species, resistance mechanisms, and affected cellular antimicrobial targets. Their work identified features to distinguish between AMR and AMS strains, including a subset of features shared across species with different resistance mechanisms, which suggest shared biological signatures of resistance.

Giddey et al. investigated the adaptive responses to sublethal concentrations of rifampicin (frontline anti-TB drug). The isogenic mutant harboring the clinically relevant S531L rifampicin resistance-conferring mutation (SL) distinguished the responses that were specific to RNA polymerase β subunit- (RpoB-) binding activity of rifampicin from those that were dependent on the presence of rifampicin alone. Then, using a cell wall-enrichment strategy focussed on the cell wall proteome, they observed 253 dysregulated proteins in SL bacteria compared to 716 proteins in wild-type strains. The drug-resistant Mycobacterium smegmatis strain displayed some of the same proteomic responses observed in WT and suggests that this evidence supported the hypothesis that rifampicin exercises effects beyond RpoB-interaction alone and that mycobacteria recognize rifampicin as a signaling molecule in an RpoB-independent manner at sublethal doses. In summary, the remodeling in WT mycobacteria is mixed RpoB-independent and RpoB-dependent proteomic changes, with evidence for RpoB-independent ABC transporter down-regulation, but drug activity-based transcriptional up-regulation and two-component system down-regulation.

Fortuin and Soares give us their opinion on the overuse of antimicrobials and the rise of drug-resistant pathogens, which has become a global health threat. They also considered that understanding the mechanisms of bacterial drug resistance is of clinical significance, whether the resistance is acquired in hospital or in the community, because it plays an important role in the treatment strategy and in controlling infectious diseases. The authors described the advances in mass spectrometry-based proteomics in bacterial proteomics, as well as metabolomics analysis, focusing on bacterial drug resistance. They also commented on the advances in omics technologies over the last few decades, which now allow multi-omics studies, in order to obtain a comprehensive understanding of the biochemical alterations of pathogenic bacteria in the context of antibiotic exposure, to identify novel biomarkers to develop new drug targets, and to develop timely screening for drug susceptibility or resistance using proteomics and metabolomics.

## Author Contributions

ET-S wrote the initial draft. All authors have contributed to drafting the work and revising it critically for important intellectual content, providing the approval for publication of the content, and the agreement to be accountable for all aspects of the work in ensuring that questions related to the accuracy or integrity of any part of the work are appropriately investigated and resolved.

## Funding

This study is part of Human Disease Biomarkers Discovery Research group, UOS. The work at the Liu laboratory was financially supported with grants from the National Natural Science Foundation of China (21974002 and 22174003) and Beijing Municipal Natural Science Foundation (5202012).

## Conflict of Interest

The authors declare that the research was conducted in the absence of any commercial or financial relationships that could be construed as a potential conflict of interest.

## Publisher's Note

All claims expressed in this article are solely those of the authors and do not necessarily represent those of their affiliated organizations, or those of the publisher, the editors and the reviewers. Any product that may be evaluated in this article, or claim that may be made by its manufacturer, is not guaranteed or endorsed by the publisher.

